# Robust Meta-Model for Predicting the Likelihood of Receiving Blood Transfusion in Non-traumatic Intensive Care Unit Patients

**DOI:** 10.34133/hds.0197

**Published:** 2024-11-06

**Authors:** Alireza Rafiei, Ronald Moore, Tilendra Choudhary, Curtis Marshall, Geoffrey Smith, John D. Roback, Ravi M. Patel, Cassandra D. Josephson, Rishikesan Kamaleswaran

**Affiliations:** ^1^ Department of Computer Science, Emory University, Atlanta, GA, USA.; ^2^ Department of Biomedical Informatics, Emory University School of Medicine, Atlanta, GA, USA.; ^3^ Department of Pathology and Laboratory Medicine, Emory University School of Medicine, Atlanta, GA, USA.; ^4^ Department of Pediatrics, Emory University School of Medicine, Atlanta, GA, USA.; ^5^ Department of Oncology, The Johns Hopkins University School of Medicine, Baltimore, MD, USA.; ^6^ Cancer and Blood Disorders Institute, Johns Hopkins All Children’s Hospital, St. Petersburg, FL, USA.

## Abstract

**Background:** Blood transfusions, crucial in managing anemia and coagulopathy in intensive care unit (ICU) settings, require accurate prediction for effective resource allocation and patient risk assessment. However, existing clinical decision support systems have primarily targeted a particular patient demographic with unique medical conditions and focused on a single type of blood transfusion. This study aims to develop an advanced machine learning-based model to predict the probability of transfusion necessity over the next 24 h for a diverse range of non-traumatic ICU patients. **Methods:** We conducted a retrospective cohort study on 72,072 non-traumatic adult ICU patients admitted to a high-volume US metropolitan academic hospital between 2016 and 2020. We developed a meta-learner and various machine learning models to serve as predictors, training them annually with 4-year data and evaluating on the fifth, unseen year, iteratively over 5 years. **Results:** The experimental results revealed that the meta-model surpasses the other models in different development scenarios. It achieved notable performance metrics, including an area under the receiver operating characteristic curve of 0.97, an accuracy rate of 0.93, and an F1 score of 0.89 in the best scenario. **Conclusion:** This study pioneers the use of machine learning models for predicting the likelihood of blood transfusion receipt in a diverse cohort of critically ill patients. The findings of this evaluation confirm that our model not only effectively predicts transfusion reception but also identifies key biomarkers for making transfusion decisions.

## Introduction

Patients in the intensive care unit (ICU) frequently develop anemia or coagulopathy that is associated with adverse outcomes, such as increasing risk of life-threatening situations, thrombosis, and coronary artery diseases [1]. Postsurgical and accident-affected patients also suffer from a high risk of mortality due to severe blood loss. Transfusion of blood components is generally recommended as a clinical treatment in such scenarios. Massive blood transfusions are essential for patients with uncontrolled intraoperative hemorrhage to avoid complications. The massive blood transfusion protocol is commonly applied to trauma patients. In transfusion medicine, trauma typically refers to major physical injury or massive bleeding due to an accident or surgery. In contrast, non-traumatic blood transfusions are needed for a variety of clinical reasons that are not associated with physical injuries or trauma. The reasons include healthy blood cell deficiency, anemia, coagulopathy, and other disorders (e.g., thrombocytopenia, hemophilia, kidney or liver disease, severe infection, and sickle cell disease). However, identification of non-traumatic ICU patients requiring transfusions is more difficult than identifying traumatic patients requiring massive transfusions. Compared to all other blood products, resuscitation with red blood cell (RBC) components is most common and frequent in transfusion patients. Approximately 85 million RBC units are transfused each year worldwide, and about 15 million are annually transfused in the United States [2]. In clinical practices, physicians often make decisions for blood transfusion primarily based on a few lab-screening features of a patient, such as anemia symptoms, hemoglobin levels, and platelet count. For example, the need for RBC transfusion is mostly decided by a hemoglobin threshold level of 7 to 8 g/dl, also suggested by the American Association of Blood Banks [2]. However, in urgent scenarios of ICU, clinicians may not be able to exhaustively evaluate all markers of a patient, such as clinical history, lab values, and demographics, which can be important. Delayed infusion, improper dosage, and type of blood-products selection in transfusion may even degrade the patient’s health. Thus, devising an efficient decision-making tool is critical to optimize the treatment strategies for blood transfusion of ICU patients.

Numerous research studies on predicting RBC transfusion are well documented in the literature. The techniques used in these works vary from clinical measures [3] and standard regression analysis [4,5] to more complex machine learning methods such as neural networks [6–9] and reinforcement learning [1]. It is important to note that the majority of these prior studies were focused on the transfusion of patients undergoing specific operations, including cardiovascular surgery [10–12], head and neck surgery [13], liver transplantation [14], prostatectomy [15], and hip fracture surgery [16]. Additionally, most of the previous literature on blood transfusion prediction had incorporated patient demographics into model development [5,6,8–11,13,16–18], which may lead to biased predictions during evaluation. Fortunately, informative routinely collected laboratory tests are available to aid in the development of these models, including hemoglobin, hematocrit, platelet count, white blood cell count, creatinine, international normalized ratio, bilirubin, partial thromboplastin time (PTT). However, existing works use a small subset of these lab values in their predictive model developments. Therefore, it is imperative to perform a more generalized analysis for all kinds of non-massively bleeding ICU patients, irrespective of diagnoses and demographic variables.

**Fig. 1.**
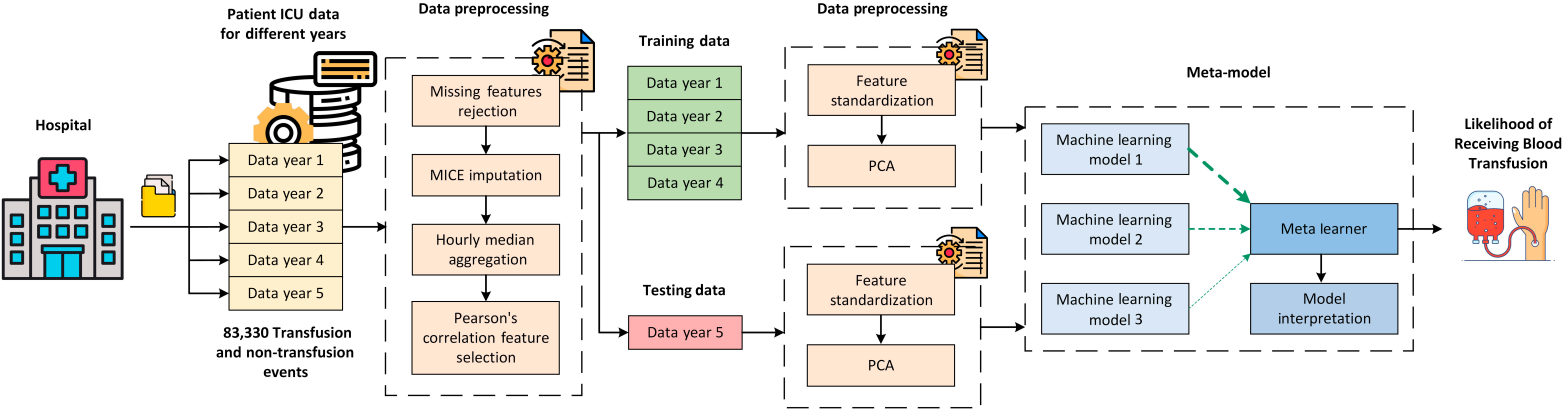
Workflow diagram of the proposed architecture. Electronic health records data collected from Emory University Hospital is preprocessed using missing features rejection, MICE imputation, aggregation, and Pearson’s correlation feature selection. One year of data is used for testing, while the other years of data are used for training. The data is then further preprocessed using feature standardization and principal component analysis (PCA) before being input into the meta-model for development, evaluation, and model interpretation.

In this study, a unique combination of parameterized machine learning-based schemes and extensive clinical features was employed to devise a decision model for predicting the likelihood of receiving blood transfusion in critical care units. This model can offer healthcare providers highly reliable support for predicting blood transfusion recipients, thereby facilitating proactive management of at-risk patients. To broaden the understanding of the rationale behind transfusion receipt and to enhance prediction efficiency, we explored different parameterized machine learning-based schemes, utilizing an extensive set of clinical features, to develop a clinical support decision system for transfusion receipt prediction in critically ill patients. The research centers on pinpointing which ICU patients will most likely receive a blood transfusion in the following 24 h. For this aim, we proposed a generalizable and interpretable meta-model capable of predicting the likelihood of receiving transfusions of various blood products, including RBC, plasma, and platelets. The general workflow for our proposed architecture can be viewed in Fig. 1.

Our contributions are as follows:

• Conduct a broad analysis on a large scale of non-traumatic critically ill patient cohorts with different medical conditions over 5 years.

• Propose a meta-model for transfusion prediction that develops generalizable knowledge of transfusion patients.

• Feature importance analysis of the meta-model to interpret reasoning behind the model’s transfusion predictions.

## Methods

### Data collection

Physiological data was continuously acquired and archived using the BedMaster (Excel Medical, Jupiter, FL) software from 150 ICU beds at Emory University Hospital (Atlanta, GA). Many clinical features were collected continuously at a sampling interval of 1 h from a given patient’s admission through to discharge. However, some were derived from the electronic health records of enrolled patients. Extracted clinical features consist of vital signs and lab values from complete blood count, hepatic, pancreatic, cardiac, arterial blood gas, and inflammation tests.

In this retrospective study, up to 24 h of data preceding transfusion initiation was used for transfused patients admitted from 2016 to 2020, containing 72,072 patient encounters. Clinical data of the 24-h timing window after the admission was considered for other non-transfused patients. Depending on the severity, each patient may undergo multiple transfusions, and thus, for every patient, clinical features were median-aggregated in their processing windows to have single entries per transfusion.

**Fig. 2.**
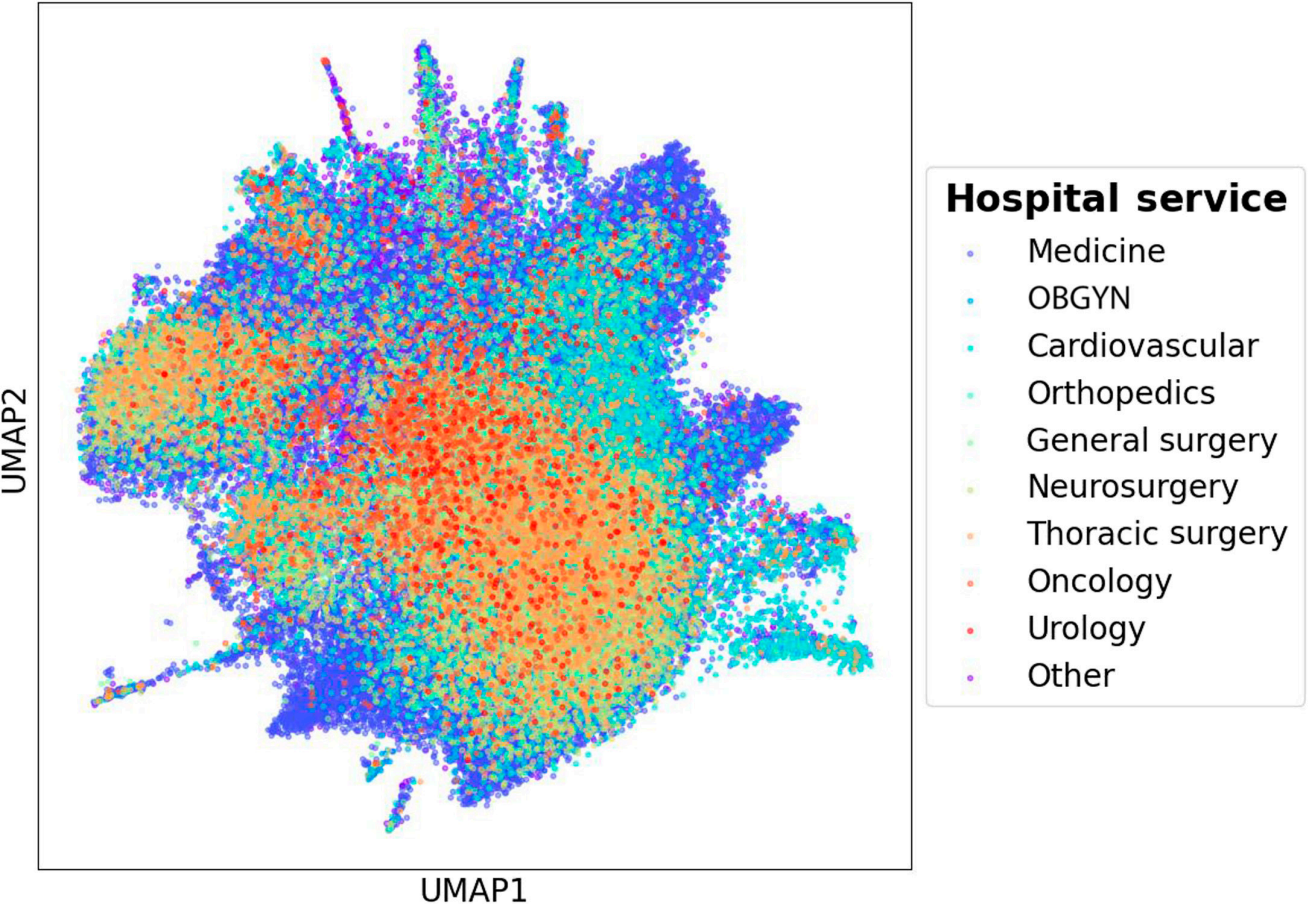
UMAP presenting all transfusion and non-transfusion events, characterized by clinical values, in 2016 to 2020 from various hospital services. Note that OBGYN refers to obstetrics and gynecology.

In this study, the Transfused cohort was created with non-traumatic patients satisfying the following inclusion criteria: (a) adult patient with age ≥ 18 years, admitted to an ICU; (b) transfused with RBC, platelets, plasma, or whole blood products; and (c) with no massive bleeding. We excluded the following patients: (a) massively transfused patients showing massive bleeding/traumatic complications by discarding those who received more than 3 transfusions in a continuous 6-h window, (b) patients with inadequate data for processing and having all the features missing, and (c) patients discharged or died after their ICU admission within 24 h, due to limited duration of physiological data available. Whereas all the adult ICU patients (≥18 years) without any blood transfusion were included in the Non-transfused group. The abovementioned exclusion criteria were also applied to the non-transfused cohort. Eventually, the study included a total of 18,314 transfused and 53,758 non-transfused encounters. Demographic distribution and clinical statistics of involved patients are summarized in Table 1. For better generalization, our study involves patients from various hospital departments and surgery sections. All transfusion and non-transfusion patients’ distribution characterized by clinical features is shown by a Uniform Manifold Approximation and Projection for Dimension Reduction (UMAP) representation in Fig. 2, where color labels depict various hospital service sections.

**Table 1.** Cohort characteristics for patients admitted to the hospital from 2016 to 2020

Characteristic	Total encounters *n* = 72,072 (100%)	Non-transfused *n* = 53,758 (74.6%)	Transfused^a^ *n* = 18,314 (25.4%)	*P* value^b^
Age, median [95% CI]	63.0 [25.0, 90.0]	62.0 [24.0, 90.0]	64.0 [26.0, 88.0]	< 0.001
Gender, *n* (%)				
Female	33,985 (47.2)	24,834 (46.2)	9,151 (50.0)	< 0.001
Male	38,087 (52.8)	28,924 (53.8)	9,163 (50.0)	
Race, *n* (%)				
African American or Black	29,833 (41.4)	22,107 (41.1)	7,726 (42.2)	0.012
Caucasian or White	36,317 (50.4)	27,263 (50.7)	9,054 (49.4)	
Other	5,922 (8.2)	4,388 (8.2)	1,534 (8.4)	
Ethnicity, *n* (%)				
Hispanic or Latino	2,226 (3.1)	1,679 (3.1)	547 (3.0)	0.303
Non-Hispanic or Latino	64,667 (89.7)	48,180 (89.6)	16,487 (90.0)	
Other	5,179 (7.2)	3,899 (7.3)	1,280 (7.0)	
Hospital service, *n* (%)				
Medicine	32,245 (44.7)	25,212 (46.9)	7,033 (38.4)	< 0.001
OBGYN	323 (0.4)	219 (0.4)	104 (0.6)	
Cardiovascular	13,416 (18.6)	10,396 (19.3)	3,020 (16.5)	
Orthopedics	1,538 (2.1)	1,088 (2.0)	450 (2.5)	
General surgery	2,417 (3.4)	1,349 (2.5)	1,068 (5.8)	
Neurosurgery	4,643 (6.4)	4,019 (7.5)	624 (3.4)	
Thoracic surgery	4,265 (5.9)	2,693 (5.0)	1,572 (8.6)	
Oncology	1,310 (1.8)	677 (1.3)	633 (3.5)	
Urology	363 (0.5)	236 (0.4)	127 (0.7)	
Other	11,552 (16.0)	7,869 (14.6)	3,683 (20.1)	
In-hospital mortality, *n* (%)	4,888 (6.8)	2,932 (5.5)	1,956 (10.7)	< 0.001
Height (cm), median [95% CI]	170.2 [149.9, 190.5]	170.2 [149.9, 190.5]	169.0 [149.9, 190.5]	< 0.001
Weight (kg), median [95% CI]	81.0 [45.6, 145.0]	82.0 [45.7, 147.4]	78.3 [45.4, 136.4]	< 0.001
Albumin, median [95% CI]	3.4 [2.0, 4.6]	3.6 [2.2, 4.7]	3.0 [1.7, 4.3]	< 0.001
BUN, median [95% CI]	19.0 [6.0, 89.0]	18.0 [6.0, 84.0]	23.0 [6.0, 100.0]	< 0.001
Creatinine, median [95% CI]	1.0 [0.5, 9.9]	1.0 [0.5, 10.0]	1.1 [0.4, 9.5]	< 0.001
Hemoglobin, median [95% CI]	10.9 [6.6, 15.9]	11.7 [8.0, 16.2]	7.8 [5.5, 13.4]	< 0.001
Lactic acid, median [95% CI]	1.5 [0.6, 7.1]	1.5 [0.6, 6.2]	1.5 [0.6, 9.0]	< 0.001
Lipase, median [95% CI]	26.0 [3.0, 465.0]	25.0 [3.0, 505.1]	27.0 [3.0, 390.8]	< 0.001
Methemoglobin, median [95% CI]	0.4 [0.1, 1.2]	0.3 [0.0, 1.0]	0.5 [0.1, 1.4]]	< 0.001
SpO^2^/FiO^2^ ratio, median [95% CI]	250.0 [96.0, 476.2]	250.0 [95.5, 476.2]	247.8 [97.0, 476.2]	< 0.001
Platelets, median [95% CI]	210.0 [44.0, 481.0]	217.0 [83.0, 459.0]	179.0 [15.0, 534.0]	< 0.001
PTT, median [95% CI]	31.2 [22.3, 108.5]	30.9 [22.3, 115.5]	31.9 [22.3, 102.6]	< 0.001

BUN, blood urea nitrogen; FiO^2^, fraction of inspired oxygen; OBGYN, obstetrics and gynecology; PTT, partial thromboplastin time; SpO^2^, peripheral blood oxygen saturation; [95% CI], 95% confidence interval. Note that the listed dynamic features, including lab values and vital signs, are based on pretransfusion data for transfused patients and postadmission data for non-transfused patients.

^a^ Transfused column has data of all patient encounters who received at least one transfusion with no massive blood transfusion protocol. However, dynamic clinical variables were presented here by considering their index transfusions only.

^b^ *P* values for gender, race, ethnicity, hospital service, and in-hospital mortality were computed using the χ^2^ test. All other *P* values were computed using the Kruskal–Wallis test.

### Data processing

In this study, a year-wise analysis was performed for patients admitted to Emory Hospital ICU over a 5-year span, from 2016 to 2020. In routinely collected lab variables and vital signs, we discarded variables missing more than 90% of values. Subsequently, a total of 43 clinical variables were selected as independent and robust features from Pearson’s cross-correlation analysis. Table S1 displays these features along with their respective units of measurement. The Multivariate Imputation by Chained Equations (MICE) algorithm was utilized to impute missing values in features, as it has demonstrated proficiency in managing high-dimensional data and complex missing data patterns [19]. Within the scope of the MICE technique, linear regression was used for the imputation of continuous variables. Subsequently, principal component analysis was employed to reduce dimensionality, mitigate noise, and simplify the dataset. We selected the number of principal components that together explain 90% of the variability within the original dataset. These selected features are subsequently utilized by the models to estimate the likelihood of a transfusion recipient. In the initial experiment, models were trained on the 2017 to 2020 datasets and then evaluated on the 2016 dataset. In order to show temporal consistency, we conducted it iteratively on an annual basis.

### Machine learning models

We utilized 5 distinct machine learning algorithms to predict the probability of necessity for blood transfusions 24 h in advance during ICU stays. These included logistic regression (LR), random forest (RF), feedforward neural networks (FNNs), support vector machines (SVMs), and XGBoost (XGB). To improve the predictive performance of the blood transfusion receipt, a meta-model was constructed, forming a stacking ensemble model grounded in the principle of stacked generalization [20,21]. This technique harnesses the collective predictive strength of various models by aggregating individual predictions into a cohesive final prediction through a meta-model. This wisdom of the crowd approach aims to enhance different predictive performance metrics with the amalgamation of multiple base models. During the implementation, we tried different combinations of the developed base models and ultimately selected the RF, SVM, and XGB as the first-level models. Each model contributed its unique predictive strengths to the ensemble, with the objective of enhancing the overall accuracy of the final prediction. We also conducted a thorough examination of various meta-learners for transfusion receipt prediction to assess their efficacy in integrating the first-level models’ predictions. LR, RF, AdaBoost, CatBoost, GradientBoosting, voting classifier, Gaussian Naïve Bayes, Choquet fuzzy integral fusion [22], dynamic staking, and deep neural networks were analyzed. The Gaussian GradientBoosting model was finally chosen as the meta-model.

To identify the optimal set of hyperparameters for the machine learning models, we undertook an extensive search that covered the most impactful parameters across the different models. Table S2 details the hyperparameters and their associated values analyzed using a grid search strategy to pinpoint the optimal hyperparameters. Our primary performance metric was the area under the receiver operating characteristic curve (AUROC). AUROC can encapsulate a more holistic view of the classification performance of a model and is not biased by the imbalanced class distribution. As a result, models with a higher AUROC potentially lead to more efficient models in the prediction of blood transfusion by maintaining the balance between specificity and sensitivity metrics. Eventually, the performance of the developed models was assessed using AUROC, accuracy, F1 score, precision, and recall.

We considered 5 unique scenarios for training and evaluating the machine learning models on a year-by-year basis. Specifically, each model was trained using data from a 4-year period and then tested on data from a subsequent, distinct hold-out year. For instance, one of the scenarios involved training the models on data collected from 2016 to 2019 and then testing them on data from 2020. This year-wise temporal splitting method is particularly suitable for our study as it better evaluates the model’s generalizability across different time periods and better reflects real-world clinical applications where models must predict outcomes in future, unseen scenarios. All the experiments were conducted on Python 3.8.8 with scikit-learn 1.3.0, utilizing an NVIDIA GeForce GTX 950M graphics card, an Intel Core i7 processor at 2.60 GHz, and 16 GB of RAM.

## Results

### Patient cohort characteristics

Table 1 contains the characteristics of the patient cohorts, particularly of ICU patients with no massive bleeding who received at least one transfusion and those who did not receive any transfusion. It can be seen that there are no significant differences between the transfused and non-transfused patients for the lactic acid and most demographic variables. However, there are significant differences for the remaining variables in the table. Although the clinical significance of these differences remains uncertain, they highlight the vital dynamics of organ function, showing the severity of critical illnesses within ICU cohorts. Patients who received a transfusion had slightly higher creatinine levels, lower lipase levels, and lower SpO_2_/FiO_2_ ratios than their non-transfused counterparts. Additionally, those who received a transfusion also had lower hemoglobin levels and lower platelet counts than those who did not receive a transfusion. This is consistent with the transfusion criteria outlined in [2]. We also analyzed in-hospital mortality rates among patients who were either transfused or not, specifically targeting those with hemoglobin levels below 7 g/dl. In this selected cohort, we observed that 208 (10.6%) patients received transfusions, whereas 28 (1%) did not. This analysis revealed that anemic patients were more likely to receive transfusions during their end-of-life care.

Out of 72,072 patient encounters between 2016 and 2020 in the study, 18,314 received transfusions, while 53,758 did not receive any. Among all years, the highest number of transfusions was noted in 2020, the COVID-affected year, with a count of 6,515. Also, the average number of transfusions received by each transfusion encounter was 1.66 in 2020. We hypothesize that COVID might be the driving factor for rapid health deterioration, leading to the increased number of transfusions during 2020.

Additionally, to reveal the correlation between hemoglobin levels and receiving blood transfusion, Fig. S2 presents a boxplot demonstrating the distribution of hemoglobin levels in both transfused and non-transfused cohorts. A Pearson’s correlation coefficient of 0.675 was obtained (*P* < 0.001). When considering 7 g/dl as a threshold for transfusion initiation, it is observed that patients with hemoglobin levels quite above this mark also received transfusions, and patients with hemoglobin less than this mark also did not get transfused. This highlights the insufficiency of relying solely on hemoglobin levels to develop an efficient transfusion decision support system.

### Performance results and analysis

**Fig. 3.**
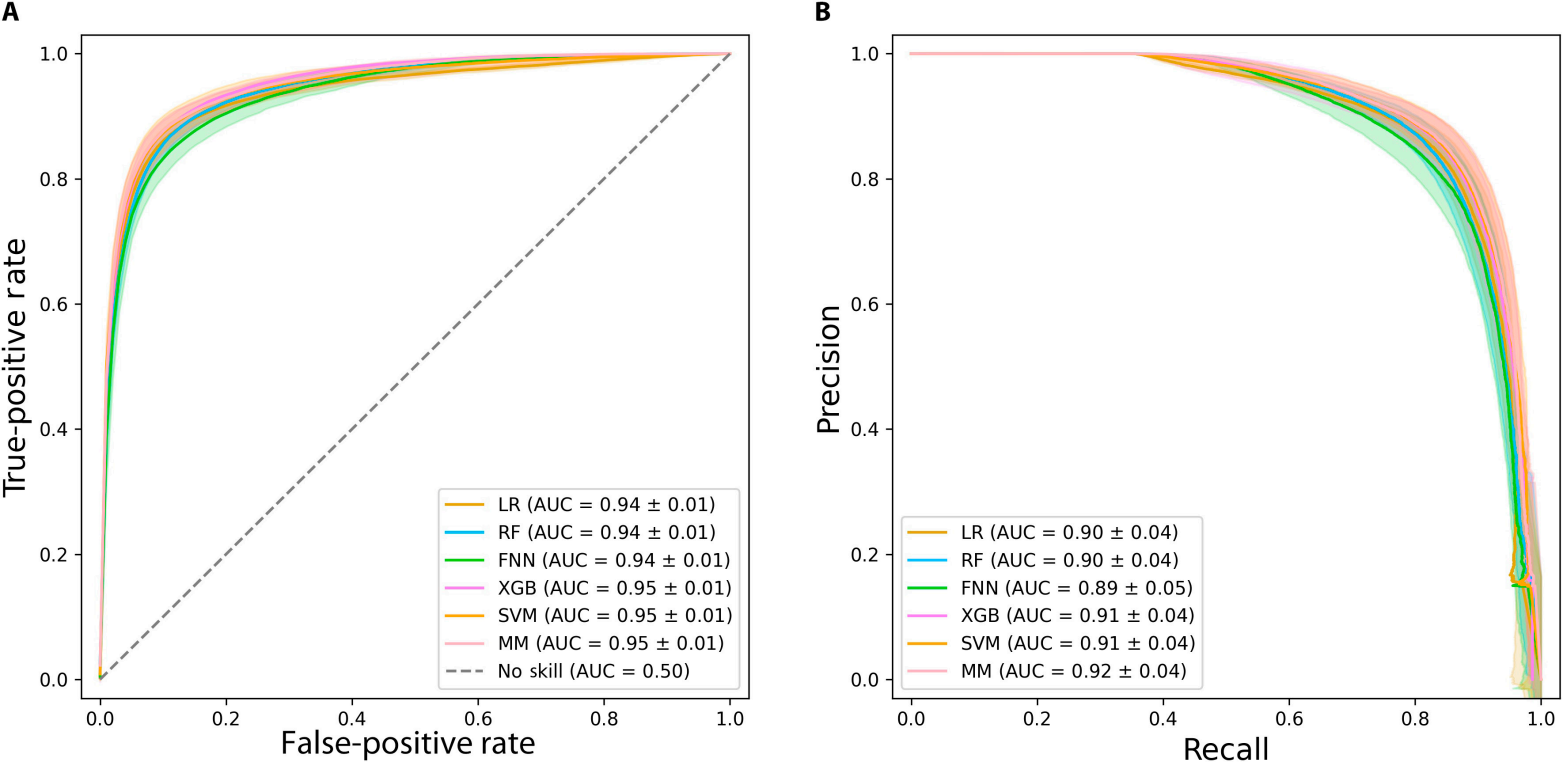
Performance metric curves of the different machine learning models. (A) ROC curves and (B) precision–recall curves of the machine learning models for transfusion receipt prediction in the 5 development scenarios. The curves are represented by a solid line indicating the mean, with the 95% confidence interval depicted as a shaded area.

The performance results of 5 different test scenarios are presented in Table 2, where the specified year denotes the evaluation period. Figure 3 shows the combined receiver operating characteristic (ROC) and precision–recall curves of the developed models for all 5 development scenarios. Of note, we calculate and plot the mean with the standard deviation of all 5 scenarios for each data point of the models. Table S3 summarizes the *P* values obtained from significant *t* test for different performance metrics of the models.

**Table 2.** Performance metrics of the developed machine learning models across different model development scenarios

Year			2016					2017					2018					2019					2020		
Metric	AUC	Acc	F1	Pre	Rec	AUC	Acc	F1	Pre	Rec	AUC	Acc	F1	Pre	Rec	AUC	Acc	F1	Pre	Rec	AUR	Acc	F1	Pre	Rec
LR	0.93	0.88	0.83	0.84	0.82	0.94	0.90	0.85	0.85	0.85	0.94	0.90	0.86	0.84	0.87	0.93	0.88	0.84	0.83	0.84	0.93	0.88	0.84	0.86	0.82
FR	0.94	0.88	0.83	0.83	0.83	0.95	0.90	0.85	0.85	0.84	0.95	0.89	0.85	0.84	0.86	0.94	0.88	0.84	0.84	0.83	0.93	0.87	0.83	0.86	0.80
FNN	0.93	0.88	0.82	0.85	0.78	0.94	0.89	0.82	0.88	0.77	0.94	0.88	0.83	0.83	0.83	0.95	0.89	0.85	0.87	0.83	0.92	0.86	0.81	0.84	0.78
XGB	0.95	0.89	0.84	0.86	0.82	0.96	0.91	0.86	0.87	0.85	0.95	0.90	0.86	0.86	0.87	0.95	0.85	0.85	0.85	0.84	0.95	0.88	0.83	0.89	0.78
SVM	0.95	0.89	0.84	0.88	0.80	0.95	0.91	0.86	0.89	0.83	0.96	0.91	0.87	0.87	0.87	0.95	0.90	0.85	0.86	0.83	0.93	0.87	0.82	0.90	0.75
MM	0.95	0.89	0.84	0.86	0.83	0.96	0.91	0.86	0.87	0.87	0.97	0.93	0.89	0.90	0.89	0.95	0.89	0.85	0.86	0.85	0.94	0.88	0.84	0.88	0.80

AUC, area under the curve; MM, meta-model

Overall, the meta-model consistently outperforms other models across various scenarios, maintaining an AUROC of at least 0.94. It exhibits well-shaped ROC and precision–recall curves, while also other models can demonstrate comparable curve shapes. Among the rest, the SVM, XGB, and FNN models register the best performance. Specifically, the SVM model excels in terms of precision across different scenarios, while the meta-model has the highest recalls. When evaluated on unseen data from the year 2018 and trained on data from other years, the meta-model achieves an impressive performance, boasting an AUROC of 0.97, an accuracy rate of 0.93, and an F1 score of 0.89. The main contribution of the meta-model can be seen in its ability to maintain high precision while improving recall. That is, it is able to identify a high proportion of the true positive cases it predicts as such, ensuring that the predictions it makes are highly reliable. At the same time, it increases the ability to capture most of the actual positive instances in the test set, effectively minimizing the chances of missing any critical positives. Figure S1 illustrates the calibration plot of the different developed models for various development scenarios. This plot reveals that all of the developed models are relatively well calibrated. In the current study, we hypothesize that the dynamic physiological markers provided by clinical labs and vital signs may have a more direct impact on receiving transfusion than the diagnosis of diseases and static demographics. Furthermore, incorporating static demographics and diagnoses into models may inadvertently introduce bias, particularly affecting minority groups [23]. Thus, we argue that excluding these variables enhances the models’ potential for fairness and generalization and allows for an improved balance between model performance, generalizability, and fairness.

**Fig. 4.**
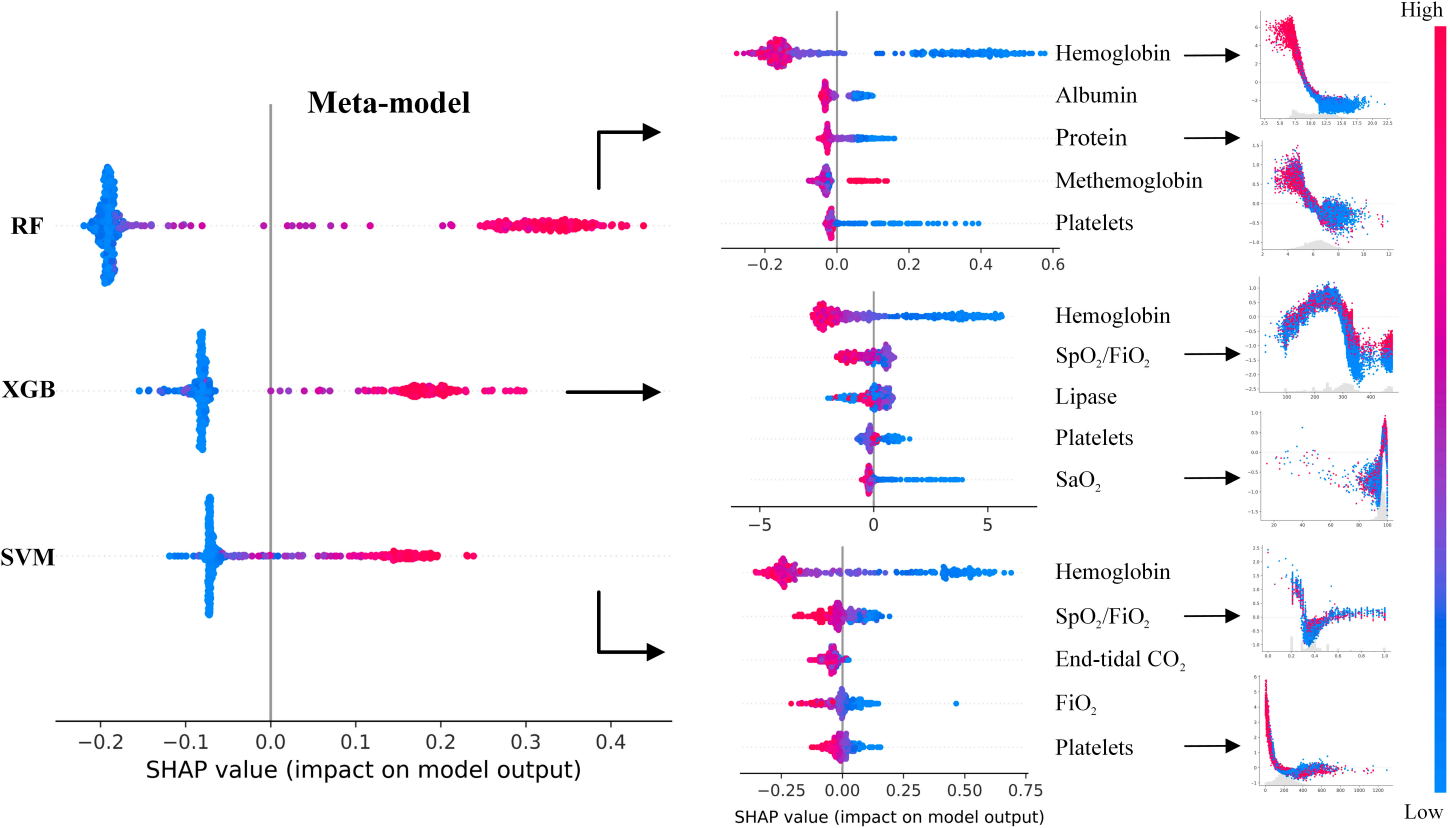
SHAP panel for the meta-model developed on the 2020 dataset.

Figure 4 presents the hierarchical SHapley Additive exPlanations (SHAP) panel of the meta-model evaluated on the 2020 data [24,25]. It offers valuable insight into how the meta-model relies on its base models to predict the necessity of a transfusion for a given patient. Notably, the prediction output from the RF algorithm stands out as the most influential model affecting the meta-model’s decisions. The second column of the panel further delineates the impact of the top 5 features within each of the 3 base models on their final predictions. Across the board, hemoglobin and platelets emerge as the most influential features in the individual machine learning models and, subsequently, the overarching meta-model. Additionally, the SHAP scatter plots provide a visual representation of the influence exerted by different features on specific predictions, illustrating both the magnitude and direction of that influence. Although the provided SHAP panel helps explain the contribution of each feature and base model, the interactions between features and the meta-model’s decision-making process may not be fully transparent. It should be noted that the SHAP panel for the meta-model, when evaluated across different years, exhibited largely similar patterns, with only minor variations. The 2020 scenario was visualized arbitrarily as an example.

## Discussion

This study aims to develop a reliable meta-model for predicting transfusion recipients, with the potential to improve patient outcomes and increase operational efficiency by revealing feature correlations that may have been overlooked or are challenging to incorporate in human decision-making. The developed model demonstrated superior performance across various training scenarios, with a full year’s data utilized for evaluation. The ability to analyze the underlying reasons behind the meta-model’s decision-making using its base models and patient features offers better communication with healthcare providers and builds trust. By enabling healthcare providers to predict transfusion recipients, this model can allow for proactive management of patients at risk, potentially improving recovery rates and reducing complications due to delayed transfusions. Additionally, improved predictive capabilities can streamline hospital operations, from optimizing blood supply management to planning staffing and procedural logistics more efficiently.

By unveiling unique complex patterns in physiological data and clinical indicators, the developed models estimate the likelihood of receiving blood transfusion among non-traumatic ICU patients well in advance. This predictive capability can be helpful in several aspects. By pinpointing those patients who are likely to receive transfusions within the next 24 h, healthcare providers can conduct further investigations and prioritize and streamline transfusion processes, making them more efficient and targeted. While the performance gains demonstrated by our ensemble model, highlighted by the significant *t* tests in Table S3, may appear significant and modest for different performance metrics, their practical implications in clinical settings are substantial. Small improvements in timely identification and increased monitoring can help to avoid the administration of unnecessary transfusions, which, in turn, reduces the risk of transfusion-related complications. This also can contribute to streamlining hospital operations, from optimizing blood supply management to planning staffing and procedural logistics more efficiently. The end result is an improvement in patient outcomes through the judicious use of medical interventions and resources, underscored by a clinical decision support data-driven approach to patient care.

Currently, the proposed study is limited to predicting the reception of blood transfusions only. Despite this limitation, the developed clinical decision support system represents a pioneering effort in predicting and issuing initial alerts for the general likelihood of receiving different types of transfusion in ICU patients with a wide range of medical conditions. The capacity to utilize a vast array of heterogeneous training data makes the algorithms more robust in the face of incomplete, noisy ICU data, and simulating different “use cases” to refine parameters is a crucial step in addressing the unique challenges associated with ICU research. After determining the necessity for a blood transfusion, clarity on the type of transfusion is crucial since different blood products are administered for various indications. As such, important next steps include extending the decision-making model’s output to encompass not only an estimation of blood transfusion receipt but also the prediction of the specific type of blood product. Additionally, integrating the prediction of the volume and rate of transfusion into these models could be beneficial. To address the limitations of SHAP analysis in fully explaining model decision-making, integrating more advanced interpretability techniques such as Counterfactual Explanations to highlight input changes that would alter predictions, Anchor Explanations to provide clear if-then rules for stable predictions, and exploring causal inference models can be investigated. The next phases of this research will involve analyzing patients’ longitudinal data and conducting a prospective study. This will enable the deployment of the best-performing model in real ICU settings and allow for its performance to be enhanced through iterative optimizations. It should also be noted that we did not come across any instances of individuals refusing transfusions for reasons such as religious beliefs in the current study. However, such cases, though possibly rare, could exist and represent outliers or sources of error that are important to consider when developing and evaluating predictive models.

**Fig. 5.**
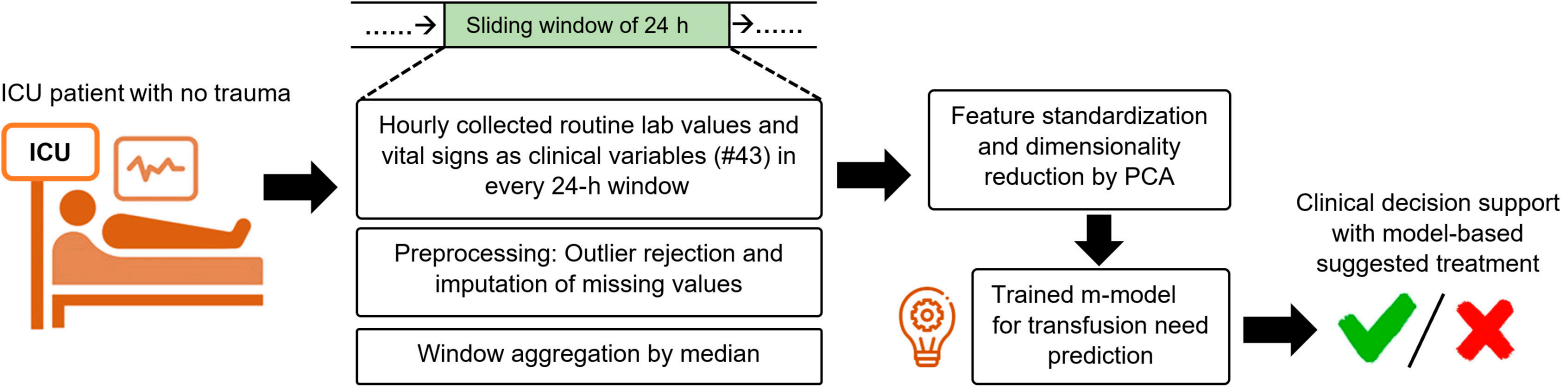
A use-case scenario of the developed meta-model includes collecting routine lab values and vital signs in a 24-h sliding window. This data is then processed through a preprocessing workflow, preparing it as input for machine learning models. Overall, a complex, critically ill, non-trauma patient poses a clinical decision for the reception of blood transfusion. The primary objective is to employ a robust machine learning model, trained on historical real-world data, to predict the clinically relevant likelihood of receiving a blood transfusion. This prediction aims to aid clinicians in enhancing their decision-making process.

When considering the accuracy of human decisions without machine learning methods, we believe that not relying on comprehensive potential features and the inability to decipher their complex inter-relations by humans may result in inappropriate transfusion decisions, specifically in non-traumatic patients. Hence, we expect that our machine learning-driven study could be utilized prospectively for clinical management and future research. A use-case scenario for deploying the proposed workflow as a clinical decision support system in the ICU settings for providing real-time predictions is shown in Fig. 5.

In this study, we developed machine learning-based prediction models for identifying critical care patients most likely to receive blood transfusions. For this aim, a unique combination of clinical features and parameterized models were explored and established. The utilization of pretransfusion laboratory values and vital signs as features had been instrumental in the development of these models. The emphasis was placed on creating a meta-learner that was not only generalizable across different patient populations but also offered clear interpretative value in its predictions regarding transfusion necessities. Our dataset consisted of a comprehensive array of transfusion-related events from over 70,000 adult patient encounters representing a broad spectrum of medical conditions, all of whom were treated at the Emory University Hospital. However, our model needs to be cross-validated with other hospitals for more generalization. Hence, future endeavors will aim to validate extensively and integrate these models into clinical workflows and assess their effectiveness on a broader scale, with the ultimate goal of refining and personalizing care in critical settings.
