# Supplementary material for: Robust Meta-Model for Predicting the Likelihood of Receiving Blood Transfusion in Non-traumatic Intensive Care Unit Patients
Source: Health Data Sci. 2024 Nov 6;4:0197. doi: 10.34133/hds.0197 (PMC11538953; doi:10.34133/hds.0197)
Supplement: Supplementary 1 — Figs. S1 and S2 Tables S1 to S4 [file hds.0197.f1.pdf]

# Robust Meta-Model for Predicting the Likelihood of Receiving Blood Transfusion in Non-traumatic ICU Patients

Alireza Rafiei[1*], Ronald Moore[1], Tilendra Choudhary[2], Curtis Marshall[2], Geoffrey Smith[3], John D. Roback[3], Ravi M. Patel[4], Cassandra D. Josephson[3,5,6], and Rishikesan Kamaleswaran[2]

[1]Department of Computer Science, Emory University, Atlanta, Georgia, USA.
[2]Department of Biomedical Informatics, Emory University School of Medicine, Atlanta, Georgia, USA.
[3]Department of Pathology and Laboratory Medicine, Emory University School of Medicine, Atlanta, Georgia, USA
[4]Department of Pediatrics, Emory University School of Medicine, Atlanta, Georgia, USA
[5]Department of Oncology, The Johns Hopkins University School of Medicine, Baltimore, Maryland, USA.
[6]Cancer and Blood Disorders Institute, Johns Hopkins All Children's Hospital, St. Petersburg, Florida, USA.
[*]Address correspondence to: alireza.rafiei@emory.edu

**Abstract**

**Background:** Blood transfusions, crucial in managing anemia and coagulopathy in ICU settings, require accurate prediction for effective resource allocation and patient risk assessment. However, existing clinical decision support systems have primarily targeted a particular patient demographic with unique medical conditions and focused on a single type of blood transfusion. This study aims to develop an advanced machine learning-based model to predict the probability of transfusion necessity over the next 24 hours for a diverse range of non-traumatic ICU patients.

**Methods:** We conducted a retrospective cohort study on 72,072 non-traumatic adult ICU patients admitted to a high-volume US metropolitan academic hospital between 2016 and 2020. We developed a meta-learner and various machine learning models to serve as predictors, training them annually with four-year data and evaluating on the fifth, unseen year, iteratively over five years.

**Results:** The experimental results revealed that the meta-model surpasses the other models in different development scenarios. It achieved notable performance metrics, including an Area

Under the Receiver Operating Characteristic (AUROC) curve of 0.97, an accuracy rate of 0.93, and an F1-score of 0.89 in the best scenario.

**Conclusion:** This study pioneers the use of machine learning models for predicting the likelihood of blood transfusion receipt in a diverse cohort of critically ill patients. The findings of this evaluation confirm that our model not only effectively predicts transfusion reception but also identifies key biomarkers for making transfusion decisions.

**Keywords:** Blood Transfusion, Intensive Care Unit, Machine Learning, Electronic Health Record, Clinical Decision Support System

# 1 Introduction

Patients in the intensive care unit (ICU) frequently develop anemia or coagulopathy that is associated with adverse outcomes, such as increasing risk of life-threatening situations, thrombosis, and coronary artery diseases [1]. Post-surgical and accident-affected patients also suffer from a high risk of mortality due to severe blood loss. Transfusion of blood components is generally recommended as a clinical treatment in such scenarios. Massive blood transfusions (MTs) are essential for patients with uncontrolled intraoperative hemorrhage to avoid complications. The MT protocol (MTP) is commonly applied to trauma patients. In transfusion medicine, *Trauma* typically refers to major physical injury or massive bleeding due to an accident or surgery. In contrast, non-traumatic blood transfusions are needed for a variety of clinical reasons that are not associated with physical injuries or trauma. The reasons include healthy blood cell deficiency, anemia, coagulopathy, and other disorders (e.g., thrombocytopenia, hemophilia, kidney or liver disease, severe infection, and sickle cell disease). However, identification of non-traumatic ICU patients requiring transfusions is more difficult than identifying traumatic patients requiring massive transfusions. Compared to all other blood products, resuscitation with red blood cell (RBC) components is most common and frequent in transfusion patients. Approximately 85 million RBC units are transfused each year worldwide, and about 15 million are annually transfused in the United States [2]. In clinical practices, physicians often make decisions for blood transfusion primarily based on a few lab-screening features of a patient, such as anemia symptoms, hemoglobin levels, and platelet count. For example, the need for RBC transfusion is mostly decided by a hemoglobin threshold level of 7 to 8 g/dL, also suggested by the American Association of Blood Banks (AABB) [2]. However, in urgent scenarios of ICU, clinicians may not be able to exhaustively evaluate all markers of a patient, such as clinical history, lab values, and demographics, which can be important. Delayed infusion, improper dosage and type of blood-products selection in transfusion may even degrade the patient's health. Thus, devising an efficient decision-making tool is critical to optimize the treatment strategies for blood transfusion of ICU patients.

Numerous research studies on predicting RBC transfusion are well-documented in the literature. The techniques used in these works vary from clinical measures [3] and standard regression analysis [4, 5] to more complex machine learning methods such as neural networks [6–9] and reinforcement learning [1]. It is important to note that the majority of these prior studies were focused on the transfusion of patients undergoing specific operations, including cardiovascular surgery [10–12], head

and neck surgery [13], liver transplantation [14], prostatectomy [15], and hip fracture surgery [16]. Additionally, most of the previous literature on blood transfusion prediction had incorporated patient demographics into model development [5, 6, 8–11, 13, 16–18], which may lead to biased predictions during evaluation. Fortunately, informative routinely collected laboratory tests are available to aid in the development of these models, including hemoglobin, hematocrit, platelet count, white blood cell count, creatinine, international normalized ratio (INR), bilirubin, partial thromboplastin time (PTT). However, existing works use a small subset of these lab values in their predictive model developments. Therefore, it is imperative to perform a more generalized analysis for all kinds of non-massively bleeding ICU patients, irrespective of diagnoses and demographic variables.

In this study, a unique combination of parameterized machine learning-based schemes and significantly comprehensive clinical features was employed to devise a decision model for predicting the likelihood of receiving blood transfusion in critical care units. This model can offer healthcare providers highly reliable support for predicting blood transfusion recipients, thereby facilitating proactive management of at-risk patients. To broaden the understanding of the rationale behind transfusion receipt and to enhance prediction efficiency, we explored different parameterized machine learning-based schemes, utilizing an extensive set of clinical features, to develop a clinical support decision system for transfusion receipt prediction in critically ill patients. The research centers on pinpointing which ICU patients will most likely receive a blood transfusion in the following 24 hours. For this aim, we proposed a generalizable and interpretable meta-model capable of predicting the likelihood of receiving transfusions of various blood products, including RBC, Plasma, and Platelets. The general workflow for our proposed architecture can be viewed in Figure 1.

Our contributions are as follows:

- Conduct a broad analysis on a large scale of non-traumatic critically ill patient cohorts with different medical conditions over five years.
- Propose a meta-model for transfusion prediction that develops generalizable knowledge of transfusion patients.
- Feature importance analysis of the meta-model to interpret reasoning behind the model's transfusion predictions.

# 2 Methods

## 2.1 Data Collection

Physiological data was continuously acquired and archived using the BedMaster (Excel Medical, Jupiter, FL) software from 150 ICU beds at Emory University Hospital (Atlanta, GA). Many clinical features were collected continuously at a sampling interval of 1-hour from a given patient's admission through to discharge. However, some were derived from the electronic health records of enrolled patients. Extracted clinical features consist of vital signs and lab values from complete blood count (CBC), hepatic, pancreatic, cardiac, arterial blood gas (ABG), and inflammation tests.

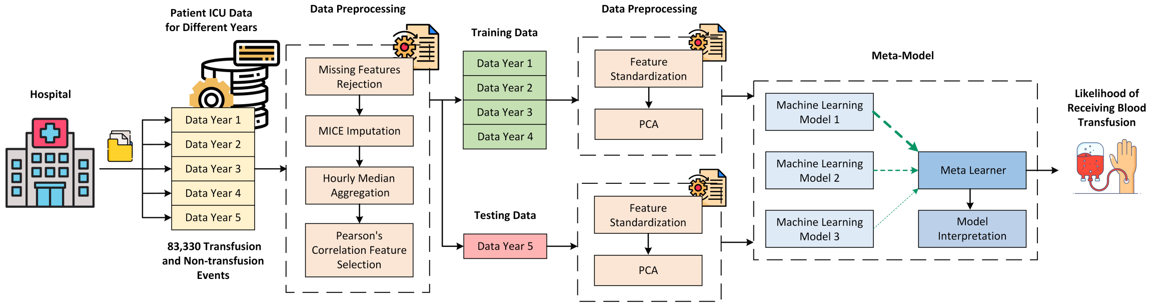


Figure 1: Workflow diagram of the proposed architecture. Electronic health records data collected from Emory University Hospital is preprocessed using missing features rejection, MICE imputation, aggregation, and Pearson's correlation feature selection. One year of data is used for testing, while the other years of data are used for training. The data is then further preprocessed using feature standardization and principal component analysis before being input into the meta-model for development, evaluation, and model interpretation.

In this retrospective study, up to 24 hours of data preceding transfusion initiation was used for transfused patients admitted from 2016 to 2020, containing 72,072 patient encounters. Clinical data of the 24-hour timing window after the admission was considered for other non-transfused patients. Depending on the severity, each patient may undergo multiple transfusions, and thus, for every patient, clinical features were median-aggregated in their processing windows to have single entries per transfusion.

In this study, the *Transfused* cohort was created with non-traumatic patients satisfying the following inclusion criteria: 1) adult patient with age$\geq$18 years, admitted to an ICU, 2) transfused with RBC, platelets, plasma, or whole blood products; and 3) with no massive bleeding. We excluded the following patients: 1) massively transfused patients showing massive bleeding/traumatic complications by discarding those who received more than three transfusions in a continuous 6-hour window, 2) patients with inadequate data for processing and having all the features missing, and 3) patients discharged or died after their ICU admission within 24 hours, due to limited duration of physiological data available. Whereas all the adult ICU patients ($\geq$18 years) without any blood transfusion were included in the *Non-transfused* group. The abovementioned exclusion criteria were also applied to the non-transfused cohort. Eventually, the study included a total of 18,314 transfused and 53,758 non-transfused encounters. Demographic distribution and clinical statistics of involved patients are summarized in Table 1. For better generalization, our study involves patients from various hospital departments and surgery sections. All transfusion and non-transfusion patients' distribution characterized by clinical features is shown by a Uniform Manifold Approximation and Projection for Dimension Reduction (UMAP) representation in Figure 2, where color labels depict various hospital service sections.

## 2.2 Data Processing

In this study, a year-wise analysis was performed for patients admitted to Emory Hospital ICU over a five-year span, from 2016 to 2020. In routinely collected lab variables and vital signs, we discarded

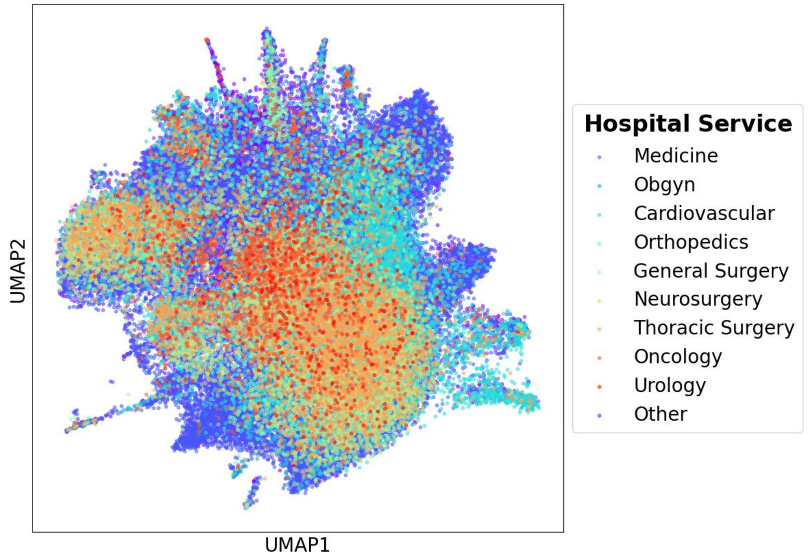


Figure 2: UMAP presenting all transfusion and non-transfusion events, characterized by clinical values, in 2016-2020 from various hospital services. Note that OBGYN refers to Obstetrics and Gynecology.

variables missing more than 90% of values. Subsequently, a total of 43 clinical variables were selected as independent and robust features from Pearson's cross-correlation analysis. Table S1 displays these features along with their respective units of measurement. The Multivariate Imputation by Chained Equations (MICE) algorithm was utilized to impute missing values in features, as it has demonstrated proficiency in managing high-dimensional data and complex missing data patterns [19]. Within the scope of the MICE technique, linear regression was used for the imputation of continuous variables. Subsequently, principal component analysis (PCA) was employed to reduce dimensionality, mitigate noise, and simplify the dataset. We selected the number of principal components that together explain 90% of the variability within the original dataset. These selected features are subsequently utilized by the models to estimate the likelihood of a transfusion recipient. In the initial experiment, models were trained on the 2017 to 2020 datasets and then evaluated on the 2016 dataset. In order to show temporal consistency, we conducted it iteratively on an annual basis.

## 2.3 Machine Learning Models

We utilized five distinct machine learning algorithms to predict the probability of necessity for blood transfusions 24 hours in advance during ICU stays. These included logistic regression (LR), random forest (RF), feedforward neural networks (FNN), support vector machines (SVM), and XGBoost (XGB). To improve the predictive performance of the blood transfusion receipt, a meta-model was constructed, forming a stacking ensemble model grounded in the principle of stacked generalization [20, 21]. This technique harnesses the collective predictive strength of various models by aggregating individual predictions into a cohesive final prediction through a meta-model. This wisdom of the crowd approach aims to enhance different predictive performance metrics with the amalgamation of

multiple base models. During the implementation, we tried different combinations of the developed base models and ultimately selected the RF, SVM, and XGB as the first-level models. Each model contributed its unique predictive strengths to the ensemble, with the objective of enhancing the overall accuracy of the final prediction. We also conducted a thorough examination of various meta-learners for transfusion receipt prediction to assess their efficacy in integrating the first-level models' predictions. LR, RF, AdaBoost, CatBoost, GradientBoosting, voting classifier, Gaussian Naïve Bayes, Choquet fuzzy integral fusion [22], dynamic staking, and deep neural networks were analyzed. The Gaussian GradientBoosting model was finally chosen as the meta-model.

To identify the optimal set of hyperparameters for the machine learning models, we undertook an extensive search that covered the most impactful parameters across the different models. Table S2 details the hyperparameters and their associated values analyzed using a grid search strategy to pinpoint the optimal hyperparameters. Our primary performance metric was the area under the receiver operating characteristic curve (AUROC). AUROC can encapsulate a more holistic view of the classification performance of a model and is not biased by the imbalanced class distribution. As a result, models with a higher AUROC potentially lead to more efficient models in the prediction of blood transfusion by maintaining the balance between specificity and sensitivity metrics. Eventually, the performance of the developed models was assessed using AUROC, accuracy, F1-score, precision, and recall.

We considered five unique scenarios for training and evaluating the machine learning models on a year-by-year basis. Specifically, each model was trained using data from a four-year period and then tested on data from a subsequent, distinct hold-out year. For instance, one of the scenarios involved training the models on data collected from 2016 to 2019 and then testing them on data from 2020. This year-wise temporal splitting method is particularly suitable for our study as it better evaluates the model's generalizability across different time periods and better reflects real-world clinical applications where models must predict outcomes in future, unseen scenarios. All the experiments were conducted on Python 3.8.8 with scikit-learn 1.3.0, utilizing an NVIDIA GeForce GTX 950M graphics card, an Intel Core i7 processor at 2.60GHz, and 16GB of RAM.

# 3 Results and Discussion

## 3.1 Patient Cohort Characteristics

Table 1 contains the characteristics of the patient cohorts, particularly of ICU patients with no massive bleeding who received at least one transfusion and those who did not receive any transfusion. It can be seen that there are no significant differences between the transfused and non-transfused patients for the lactic acid and most demographic variables. However, there are significant differences for the remaining variables in the table. Although the clinical significance of these differences remains uncertain, they highlight the vital dynamics of organ function, showing the severity of critical illnesses within ICU cohorts. Patients who received a transfusion had slightly higher creatinine levels, lower lipase levels, and lower $SpO_2/FiO_2$ ratios than their non-transfused counterparts. Additionally, those who received a transfusion also had lower hemoglobin levels and lower platelet counts than

Table 1: Cohort characteristics for patients admitted to the hospital from 2016 to 2020.

| **Characteristic** | **Total encounters n = 72072 (100%)** | **Non-transfused n = 53758 (74.6%)** | **†Transfused n = 18314 (25.4%)** | ***p-value** |
|---|---|---|---|---|
| Age, median [95% CI] | 63.0 [25.0, 90.0] | 62.0 [24.0, 90.0] | 64.0 [26.0, 88.0] | <0.001 |
| Gender, n (%) | | | | |
| Female | 33985 (47.2) | 24834 (46.2) | 9151 (50.0) | <0.001 |
| Male | 38087 (52.8) | 28924 (53.8) | 9163 (50.0) | |
| Race, n (%) | | | | |
| African American or Black | 29833 (41.4) | 22107 (41.1) | 7726 (42.2) | 0.012 |
| Caucasian or White | 36317 (50.4) | 27263 (50.7) | 9054 (49.4) | |
| Other | 5922 (8.2) | 4388 (8.2) | 1534 (8.4) | |
| Ethnicity, n (%) | | | | |
| Hispanic or Latino | 2226 (3.1) | 1679 (3.1) | 547 (3.0) | 0.303 |
| Non-Hispanic or Latino | 64667 (89.7) | 48180 (89.6) | 16487 (90.0) | |
| Other | 5179 (7.2) | 3899 (7.3) | 1280 (7.0) | |
| Hospital Service, n (%) | | | | |
| Medicine | 32245 (44.7) | 25212 (46.9) | 7033 (38.4) | <0.001 |
| OBGYN | 323 (0.4) | 219 (0.4) | 104 (0.6) | |
| Cardiovascular | 13416 (18.6) | 10396 (19.3) | 3020 (16.5) | |
| Orthopedics | 1538 (2.1) | 1088 (2.0) | 450 (2.5) | |
| General Surgery | 2417 (3.4) | 1349 (2.5) | 1068 (5.8) | |
| Neurosurgery | 4643 (6.4) | 4019 (7.5) | 624 (3.4) | |
| Thoracic Surgery | 4265 (5.9) | 2693 (5.0) | 1572 (8.6) | |
| Oncology | 1310 (1.8) | 677 (1.3) | 633 (3.5) | |
| Urology | 363 (0.5) | 236 (0.4) | 127 (0.7) | |
| Other | 11552 (16.0) | 7869 (14.6) | 3683 (20.1) | |
| In-Hospital Mortality, n (%) | 4888 (6.8) | 2932 (5.5) | 1956 (10.7) | <0.001 |
| Height (cm), median [95% CI] | 170.2 [149.9, 190.5] | 170.2 [149.9, 190.5] | 169.0 [149.9, 190.5] | <0.001 |
| Weight (kg), median [95% CI] | 81.0 [45.6, 145.0] | 82.0 [45.7, 147.4] | 78.3 [45.4, 136.4] | <0.001 |
| Albumin, median [95% CI] | 3.4 [2.0, 4.6] | 3.6 [2.2, 4.7] | 3.0 [1.7, 4.3] | <0.001 |
| BUN, median [95% CI] | 19.0 [6.0, 89.0] | 18.0 [6.0, 84.0] | 23.0 [6.0, 100.0] | <0.001 |
| Creatinine, median [95% CI] | 1.0 [0.5, 9.9] | 1.0 [0.5, 10.0] | 1.1 [0.4, 9.5] | <0.001 |
| Hemoglobin, median [95% CI] | 10.9 [6.6, 15.9] | 11.7 [8.0, 16.2] | 7.8 [5.5, 13.4] | <0.001 |
| Lactic Acid, median [95% CI] | 1.5 [0.6, 7.1] | 1.5 [0.6, 6.2] | 1.5 [0.6, 9.0] | <0.001 |
| Lipase, median [95% CI] | 26.0 [3.0, 465.0] | 25.0 [3.0, 505.1] | 27.0 [3.0, 390.8] | <0.001 |
| Methemoglobin, median [95% CI] | 0.4 [0.1, 1.2] | 0.3 [0.0, 1.0] | 0.5 [0.1, 1.4]] | <0.001 |
| $SpO_2/FiO_2$ Ratio, median [95% CI] | 250.0 [96.0, 476.2] | 250.0 [95.5, 476.2] | 247.8 [97.0, 476.2] | <0.001 |
| Platelets, median [95% CI] | 210.0 [44.0, 481.0] | 217.0 [83.0, 459.0] | 179.0 [15.0, 534.0] | <0.001 |
| PTT, median [95% CI] | 31.2 [22.3, 108.5] | 30.9 [22.3, 115.5] | 31.9 [22.3, 102.6] | <0.001 |

Abbreviations used – BUN: blood urea nitrogen, $FiO_2$: fraction of inspired oxygen, OBGYN: obstetrics and gynecology, PTT: partial prothrombin time, $SpO_2$: peripheral blood oxygen saturation, [95% CI]: 95 percent confidence interval. Note that the listed dynamic features, including lab values and vital signs, are based on pre-transfusion data for transfused patients and post-admission data for non-transfused patients.

* P-values for Gender, Race, Ethnicity, Hospital Service, and In-Hospital Mortality were computed using the Chi-square test. All other p-values were computed using the Kruskal-Wallis test.

† Transfused column has data of all patient encounters who received at least one transfusion with no MTP. However, dynamic clinical variables were presented here by considering their index transfusions only.

those who did not receive a transfusion. This is consistent with the transfusion criteria outlined by [2]. We also analyzed in-hospital mortality rates among patients who were either transfused or not, specifically targeting those with hemoglobin levels below 7 g/dL. In this selected cohort, we observed that 208 (10.6%) patients received transfusions, whereas 28 (1%) did not. This analysis revealed that anemic patients were more likely to receive transfusions during their End-of-Life care.

Out of 72,072 patient encounters between 2016 and 2020 in the study, 18,314 received transfusions, while 53,758 did not receive any. Among all years, the highest number of transfusions was noted in 2020, the COVID-affected year, with a count of 6515. Also, the average number of transfusions received by each transfusion encounter was 1.66 in 2020. We hypothesize that COVID might be the driving factor for rapid health deterioration, leading to the increased number of transfusions

Table 2: Performance metrics of the developed machine learning models across different model development scenarios.

| **Year** | | | **2016** | | | | | **2017** | | | | | **2018** | | | | | **2019** | | | | | **2020** | | |
|---|---|---|---|---|---|---|---|---|---|---|---|---|---|---|---|---|---|---|---|---|---|---|---|---|---|
| Metric | AUC | Acc | F1 | Pre | Rec | AUC | Acc | F1 | Pre | Rec | AUC | Acc | F1 | Pre | Rec | AUC | Acc | F1 | Pre | Rec | AUR | Acc | F1 | Pre | Rec |
| LR | 0.93 | 0.88 | 0.83 | 0.84 | 0.82 | 0.94 | 0.90 | 0.85 | 0.85 | 0.85 | 0.94 | 0.90 | 0.86 | 0.84 | 0.87 | 0.93 | 0.88 | 0.84 | 0.83 | 0.84 | 0.93 | 0.88 | 0.84 | 0.86 | 0.82 |
| FR | 0.94 | 0.88 | 0.83 | 0.83 | 0.83 | 0.95 | 0.90 | 0.85 | 0.85 | 0.84 | 0.95 | 0.89 | 0.85 | 0.84 | 0.86 | 0.94 | 0.88 | 0.84 | 0.84 | 0.83 | 0.93 | 0.87 | 0.83 | 0.86 | 0.80 |
| FNN | 0.93 | 0.88 | 0.82 | 0.85 | 0.78 | 0.94 | 0.89 | 0.82 | 0.88 | 0.77 | 0.94 | 0.88 | 0.83 | 0.83 | 0.83 | 0.95 | 0.89 | 0.85 | 0.87 | 0.83 | 0.92 | 0.86 | 0.81 | 0.84 | 0.78 |
| XGB | 0.95 | 0.89 | 0.84 | 0.86 | 0.82 | 0.96 | 0.91 | 0.86 | 0.87 | 0.85 | 0.95 | 0.90 | 0.86 | 0.86 | 0.87 | 0.95 | 0.85 | 0.85 | 0.85 | 0.84 | 0.95 | 0.88 | 0.83 | 0.89 | 0.78 |
| SVM | 0.95 | 0.89 | 0.84 | 0.88 | 0.80 | 0.95 | 0.91 | 0.86 | 0.89 | 0.83 | 0.96 | 0.91 | 0.87 | 0.87 | 0.87 | 0.95 | 0.90 | 0.85 | 0.86 | 0.83 | 0.93 | 0.87 | 0.82 | 0.90 | 0.75 |
| MM | 0.95 | 0.89 | 0.84 | 0.86 | 0.83 | 0.96 | 0.91 | 0.86 | 0.87 | 0.87 | 0.97 | 0.93 | 0.89 | 0.90 | 0.89 | 0.95 | 0.89 | 0.85 | 0.86 | 0.85 | 0.94 | 0.88 | 0.84 | 0.88 | 0.80 |

during 2020.

Additionally, to reveal the correlation between hemoglobin levels and receiving blood transfusion, Figure S2 presents a boxplot demonstrating the distribution of hemoglobin levels in both transfused and non-transfused cohorts. A Pearson's correlation coefficient of 0.675 was obtained ($p<0.001$). When considering 7 g/dL as a threshold for transfusion initiation, it is observed that patients with hemoglobin levels quite above this mark also received transfusions, and patients with hemoglobin less than this mark also did not get transfused. This highlights the insufficiency of relying solely on hemoglobin levels to develop an efficient transfusion decision support system.

## 3.2 Performance Results and Analysis

The performance results of five different test scenarios are presented in Table 2, where the specified year denotes the evaluation period. Figure 3 shows the combined receiver operating characteristic (ROC) and precision-recall curves of the developed models for all five development scenarios. Of note, we calculate and plot the mean with the standard deviation of all five scenarios for each data point of the models. Table S3 summarizes the p-values obtained from significant T-test for different performance metrics of the models.

Overall, the meta-model consistently outperforms other models across various scenarios, maintaining an AUROC of at least 0.94. It exhibites well-shaped ROC and precision-recall curves, while also other models can demonstrate comparable curve shapes. Among the rest, the SVM, XGB, and FNN models register the best performance. Specifically, the SVM model excels in terms of precision across different scenarios, while the meta-model has the highest recalls. When evaluated on unseen data from the year 2018 and trained on data from other years, the meta-model achieves an impressive performance, boasting an AUROC of 0.97, an accuracy rate of 0.93, and an F1 score of 0.89. The main contribution of the meta-model can be seen in its ability to maintain high precision while improving recall. That is, it is able to identify a high proportion of the true positive cases it predicts as such, ensuring that the predictions it makes are highly reliable. At the same time, it increases the ability to capture most of the actual positive instances in the test set, effectively minimizing the chances of missing any critical positives. Figure S1 illustrates the calibration plot of the different developed models for various development scenarios. This plot reveals that all of the developed models are relatively well-calibrated. In the current study, we hypothesize that the dynamic physiological markers provided by clinical labs and vital signs may have a more direct impact on receiving transfusion than the diagnosis of diseases, and static demographics. Furthermore, incorporating static demographics and diagnoses into models may inadvertently introduce bias, par-

ticularly affecting minority groups [23]. Thus, we argue that excluding these variables enhances the models' potential for fairness and generalization and allows for an improved balance between model performance, generalizability, and fairness.

Figure 4 presents the hierarchical SHapley Additive exPlanations (SHAP) panel of the meta-model evaluated on the 2020 data [24, 25]. It offers valuable insight into how the meta-model relies on its base models to predict the necessity of a transfusion for a given patient. Notably, the prediction output from the RF algorithm stands out as the most influential model affecting the meta-model's decisions. The second column of the panel further delineates the impact of the top five features within each of the three base models on their final predictions. Across the board, hemoglobin and platelets emerge as the most significant features in the individual machine learning models and, subsequently, the overarching meta-model. Additionally, the SHAP scatter plots provide a visual representation of the influence exerted by different features on specific predictions, illustrating both the magnitude and direction of that influence. Although the provided SHAP panel helps explain the contribution of each feature and base model, the interactions between features and the meta-model's decision-making process may not be fully transparent. It should be noted that the SHAP panel for the meta-model, when evaluated across different years, exhibited largely similar patterns, with only minor variations. The 2020 scenario was visualized arbitrarily as an example.

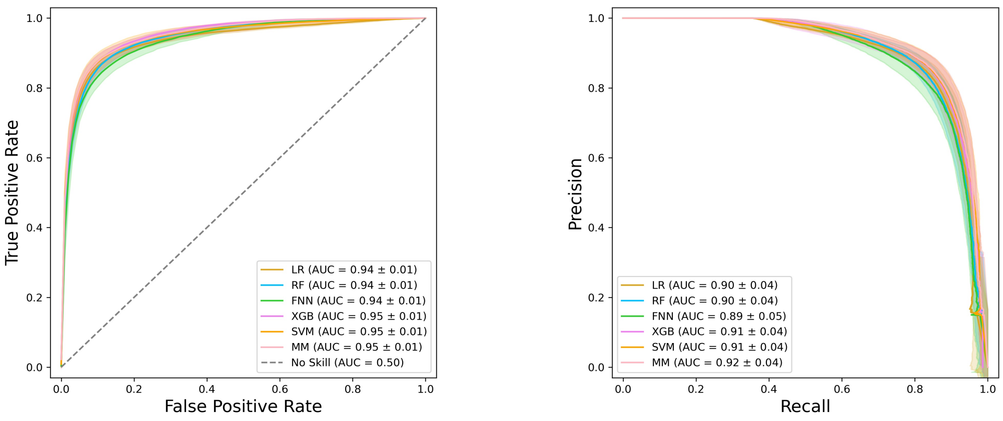


(a) (b)

Figure 3: (a) ROC curves and (b) precision-recall curves of the machine learning models for transfusion receipt prediction in the five development scenarios. The curves are represented by a solid line indicating the mean, with the 95% confidence interval depicted as a shaded area.

# 4 Discussion

This study aims to develop a reliable meta-model for predicting transfusion recipients, with the potential to improve patient outcomes and increase operational efficiency by revealing feature correlations that may have been overlooked or are challenging to incorporate in human decision-making.

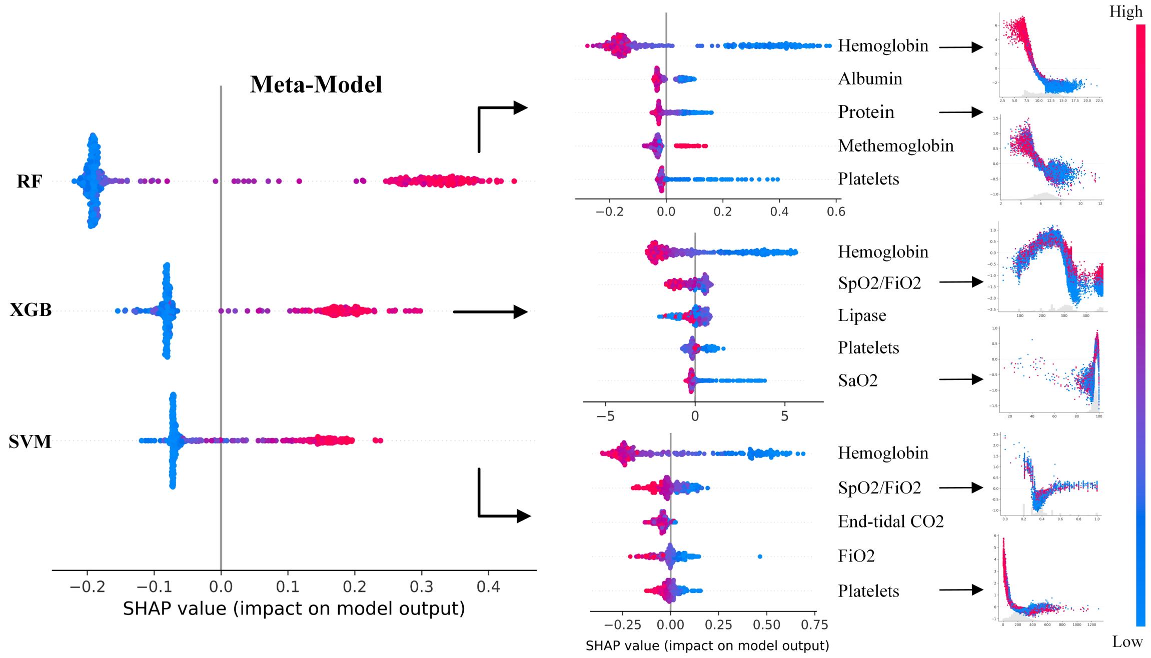


Figure 4: SHAP panel for the meta-model developed on the 2020 dataset.

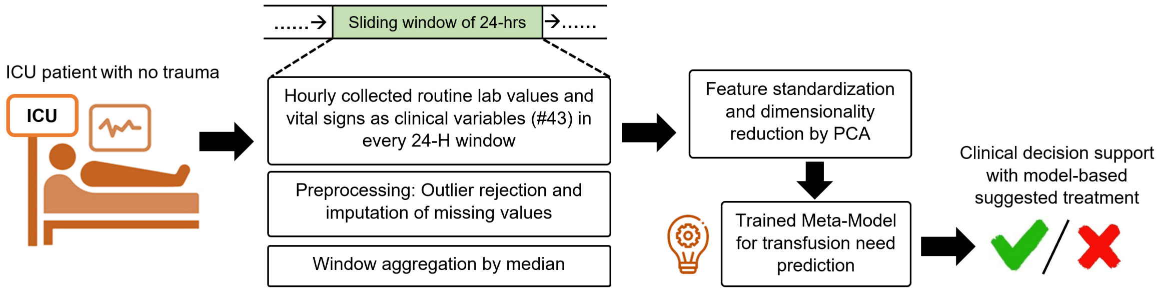


Figure 5: A use-case scenario of the developed meta-model includes collecting routine lab values and vital signs in a 24-hour sliding window. This data is then processed through a preprocessing workflow, preparing it as input for machine learning models. Overall, a complex, critically ill, non-trauma patient poses a clinical decision for the reception of blood transfusion. The primary objective is to employ a robust machine learning model, trained on historical real-world data, to predict the clinically relevant likelihood of receiving a blood transfusion. This prediction aims to aid clinicians in enhancing their decision-making process.

The developed model demonstrated significant performance across various training scenarios, with a full year's data utilized for evaluation. The ability to analyze the underlying reasons behind the meta-model's decision-making using its base models and patient features, offers better communication with healthcare providers and builds trust. By enabling healthcare providers to predict transfusion recipients, this model can allow for proactive management of patients at risk, potentially improving recovery rates and reducing complications due to delayed transfusions. Additionally, improved predictive capabilities can streamline hospital operations, from optimizing blood supply management to planning staffing and procedural logistics more efficiently.

By unveiling unique complex patterns in physiological data and clinical indicators, the developed models estimate the likelihood of receiving blood transfusion among non-traumatic ICU patients well in advance. This predictive capability can be helpful in several aspects. By pinpointing those patients who are likely to receive transfusions within the next 24 hours, healthcare providers can conduct further investigations and prioritize and streamline transfusion processes, making them more efficient and targeted. While the performance gains demonstrated by our ensemble model, highlighted by the significant T-tests in Table S3, may appear significant and modest for different performance metrics, their practical implications in clinical settings are substantial. Small improvements in timely identification and increased monitoring can help to avoid the administration of unnecessary transfusions, which, in turn, reduces the risk of transfusion-related complications. This also can contribute to streamlining hospital operations, from optimizing blood supply management to planning staffing and procedural logistics more efficiently. The end result is an improvement in patient outcomes through the judicious use of medical interventions and resources, underscored by a clinical decision support data-driven approach to patient care.

Currently, the proposed study is limited to predicting the reception of blood transfusions only. Despite this limitation, the developed clinical decision support system represents a pioneering effort in predicting and issuing initial alerts for the general likelihood of receiving different types of transfusion in ICU patients with a wide range of medical conditions. The capacity to utilize a vast array of heterogeneous training data makes the algorithms more robust in the face of incomplete, noisy ICU data, and simulating different 'use cases' to refine parameters is a crucial step in addressing the unique challenges associated with ICU research. After determining the necessity for a blood transfusion, clarity on the type of transfusion is crucial since different blood products are administered for various indications. As such, important next steps include extending the decision-making model's output to encompass not only an estimation of blood transfusion receipt but also the prediction of the specific type of blood product. Additionally, integrating the prediction of the volume and rate of transfusion into these models could be beneficial. To address the limitations of SHAP analysis in fully explaining model decision-making, integrating more advanced interpretability techniques such as Counterfactual Explanations to highlight input changes that would alter predictions, Anchor Explanations to provide clear if-then rules for stable predictions, and exploring causal inference models can be investigated. The next phases of this research will involve analyzing patients' longitudinal data and conducting a prospective study. This will enable the deployment of the best-performing model in real ICU settings and allow for its performance to be enhanced through iterative optimizations. It should also be noted that we did not come across any instances of individuals refusing

transfusions for reasons such as religious beliefs in the current study. However, such cases, though possibly rare, could exist and represent outliers or sources of error that are important to consider when developing and evaluating predictive models.

When considering the accuracy of human decisions without machine learning methods, we believe that not relying on comprehensive potential features and the inability to decipher their complex interrelations by humans may result in inappropriate transfusion decisions, specifically in non-traumatic patients. Hence, we expect that our machine learning-driven study could be utilized prospectively for clinical management and future research. A use-case scenario for deploying the proposed workflow as a clinical decision support system in the ICU settings for providing real-time predictions is shown in Figure 5.

# 5 Conclusion

In this study, we developed machine learning-based prediction models for identifying critical care patients most likely to receive blood transfusions. For this aim, a unique combination of clinical features and parameterized models were explored and established. The utilization of pre-transfusion laboratory values and vital signs as features had been instrumental in the development of these models. The emphasis was placed on creating a meta-learner that was not only generalizable across different patient populations but also offered clear interpretative value in its predictions regarding transfusion necessities. Our dataset consisted of a comprehensive array of transfusion-related events from over 70,000 adult patient encounters representing a broad spectrum of medical conditions, all of whom were treated at the Emory University Hospital. However, our model needs to be cross-validated with other hospitals for more generalization. Hence, future endeavors will aim to validate extensively and integrate these models into clinical workflows and assess their effectiveness on a broader scale, with the ultimate goal of refining and personalizing care in critical settings.

# Ethical Approval

N/A

# Data Availability

N/A

# Funding

R Kamaleswaran, T Choudhary, and C Marshall were supported by the National Institutes of Health under Award Numbers R01GM139967, R21GM151703, and R21GM148931.

# Authors' Contributions

AR, RM, and TC wrote the main manuscript and prepared all the figures and tables. AR, RM, and TC performed all machine learning and statistical analysis with GS, JR, RP, and RK providing

clinical and technical oversights. TC, CM, GS, JR, RP, CJ, and RK conceptualized the study design, provided and processed the data, and provided critical manuscript review and revision. All authors reviewed the manuscript. All authors read and approved the final manuscript.

## Conflicts of Interest

The authors declare that they have no known competing financial interests or personal relationships that could have appeared to influence the work reported in this paper.


## Acknowledgments

N/A


# Supplementary Materials

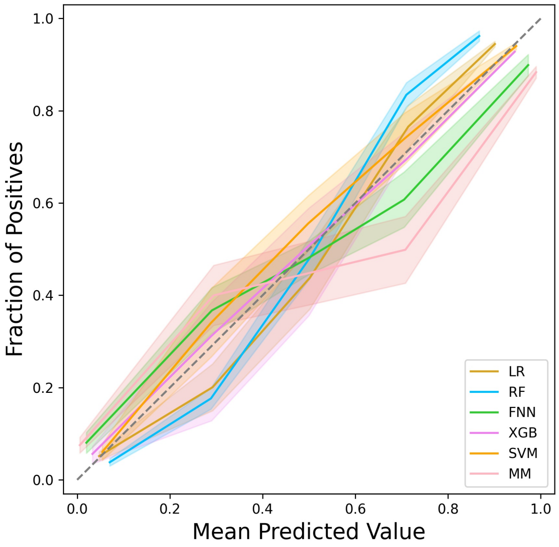


Figure S1: Calibration curves of the machine learning models using the five development scenarios.

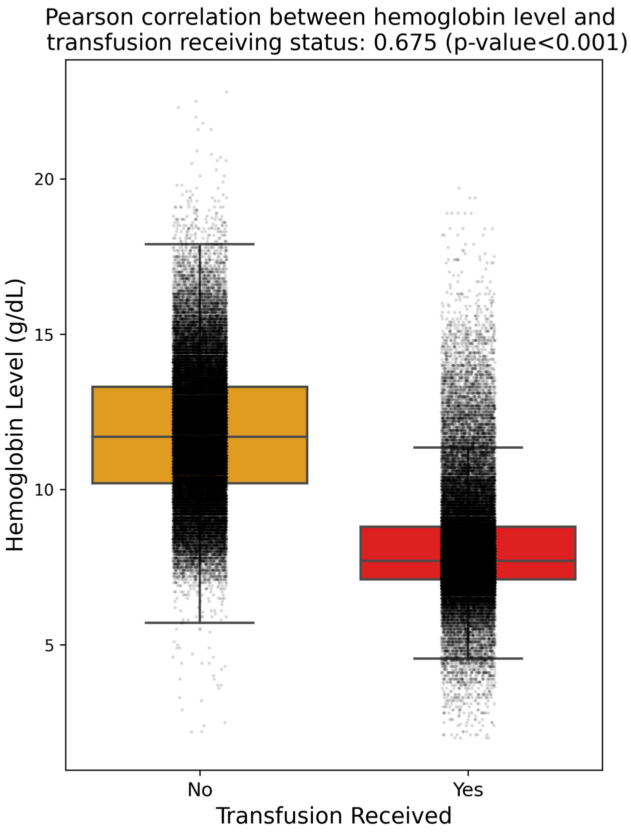


Figure S2: Distribution of hemoglobin level for transfused and non-transfused cohorts and cross-correlation of hemoglobin levels and blood transfusion decision. Each dot refers to the hemoglobin level of an ICU patient.

Table S1: List of routine clinical features selected in the study; variable names, their meanings, and measuring units.

| **Variable names and their meanings** | **Unite** |
|---|---|
| temperature: Body temperature | $^0$C |
| sbp_cuff: Cuff-based systolic blood pressure | mmHg |
| dbp_cuff: Cuff-based diastolic blood pressure | mmHg |
| pulse: Pulse rate (beats per minute) | beats per minute |
| unassisted_resp_rate: Respiratory rate | breaths per minute |
| spo2: Blood saturated oxygen concentration, SpO2 level | % |
| end_tidal_co2: End-tidal CO2 | mmHg |
| bicarb_(hco3): Bicarbonate | mmol/L |
| blood_urea_nitrogen_(bun) | mg/dL |
| chloride | mEq/L |
| creatinine | mg/dL |
| glucose | mmol/L |
| magnesium | mg/dL |
| osmolarity | mOsm/kg |
| phosphorus | mg/dL |
| potassium | mEq/L |
| sodium | mEq/L |
| hemoglobin | g/dL |
| met_hgb | g/dL |
| platelets | $\times 10^9$/L |
| white_blood_cell_count | $\times 10^9$/L |
| carboxy_hgb | % |
| alanine_aminotransferase_(alt) | U/L |
| albumin | g/L |
| alkaline_phosphatase | IU/L |
| bilirubin_direct | mg/dL |
| bilirubin_total | mg/dL |
| inr: International normalized ratio | - |
| lactic_acid | mmol/L |
| partial_prothrombin_time_(ptt) | s |
| protein | g/dL |
| lipase | U/L |
| b-type_natriuretic_peptide_(bnp) | pg/ml |
| troponin | ng/ml |
| fio2: Fraction of inspired oxygen | range: 0-1 |
| partial_pressure_of_carbon_dioxide_(paco2) | mmHg |
| partial_pressure_of_oxygen_(pao2) | mmHg |
| ph | - |
| saturation_of_oxygen_(sao2) | % |
| hemoglobin_a1c | % |
| best_map: Mean arterial pressure | mmHg |
| pf_sp: SpO2/FiO2 ratio | - |
| pf_pa: PaO2/FiO2 ratio | mmHg |

Table S2: Hyperparameter search space for tuning the models. The average AUROC for each meta-learner is provided alongside its name.

| Models | Hyperparameters | Search Space |
|---|---|---|
| RF | Number of trees in the forest | {100, 150, 200, 300, 500, 1000, 1500, 3000} |
| | Minimum sample split | {2, 4, 5, 10} |
| | Maximum depth | {5, 8, 10, 12, 15, 20} |
| SVM | Kernel type | {linear, poly, sigmoid, rbf} |
| | Regularization parameter | {0.2, 0.5, 0.8, 1, 1.5, 3, 5, 10, 25, 50} |
| XGB | Learning rate | {0.01, 0.1} |
| | Number of boosting stages | {100, 250, 500} |
| | Maximum depth | {5, 7, 12, 15} |
| | Gamma | {0, 0.1, 1} |
| FNN | Number of hidden layers | {3, 4} |
| | Number of neurons | $\{a_n = 16 + 4(n-1) \mid n \in \mathbb{Z}, 1 \leq n \leq 61\}$ |
| MM | Meta-model | {LR: 0.92, RF: 0.93, AdaBoost: 0.91, CatBoost: 0.92, GradientBoosting: 0.92, voting classifier: 0.90, Gaussian Naïve Bayes: 0.95, Choquet fuzzy integral fusion: 0.95, dynamic staking: 0.85, deep neural networks: 0.75} |

Abbreviations used – FR: random forest, SVM: support vector machine, XGB: XGBoost, FNN: feedforward neural networks, MM: meta-model

Table S3: Pairwise p-values derived from the significant T-test analysis of the performance metrics for the developed machine learning models.

| Model 1 | Model 2 | AUC | Acc | F1 | Pre | Rec |
|---|---|---|---|---|---|---|
| MM | LR | 0.008 | 0.273 | 0.290 | 0.011 | 0.673 |
| | FR | 0.094 | 0.159 | 0.159 | 0.011 | 0.409 |
| | FNN | 0.037 | 0.093 | 0.031 | 0.132 | 0.040 |
| | XGB | 0.724 | 0.249 | 0.486 | 0.451 | 0.485 |
| | SVM | 0.421 | 0.740 | 0.545 | 0.576 | 0.242 |
| SVM | LR | 0.034 | 0.739 | 0.700 | 0.003 | 0.308 |
| | FR | 0.359 | 0.222 | 0.433 | 0.003 | 0.490 |
| | FNN | 0.128 | 0.123 | 0.079 | 0.056 | 0.472 |
| | XGB | 0.471 | 0.343 | 1.000 | 0.191 | 0.542 |
| XGB | LR | 0.000 | 0.713 | 0.620 | 0.032 | 0.668 |
| | FR | 0.046 | 1.000 | 0.308 | 0.032 | 1.000 |
| | FNN | 0.019 | 0.720 | 0.039 | 0.327 | 0.131 |
| FNN | LR | 0.733 | 0.308 | 0.067 | 0.372 | 0.032 |
| | FR | 0.371 | 0.608 | 0.123 | 0.372 | 0.071 |
| FR | LR | 0.111 | 0.587 | 0.572 | 1.000 | 0.572 |

Table S4: Case study analysis of four randomly selected patients on the prediction performance and reliability of the developed meta-model and its base models. All the models predicted the labels of the first and second patients correctly. For the third patient, the RF and SVM models were unable to make accurate predictions, whereas the meta-model successfully predicted the patient's condition. Notably, the probability estimates provided by the meta-model were consistently more reliable across these cases. In the fourth scenario, despite all models failing to accurately predict the patient's actual condition, the meta-model exhibited a smaller margin of error.

| Patient Number | Actual Label | RF | XGB | SVM | MM | Predicted Label |
|---|---|---|---|---|---|---|
| 1 | 1 | 0.74 | 0.82 | 0.76 | 0.99 | 1 |
| 2 | 0 | 0.18 | 0.02 | 0.03 | 0.00 | 0 |
| 3 | 1 | 0.49 | 0.54 | 0.38 | 0.74 | 1 |
| 4 | 0 | 0.71 | 0.69 | 0.60 | 0.52 | 1 |

# References


1. Wang Y, Zhao Y, and Petzold L. Predicting the need for blood transfusion in intensive care units with reinforcement learning. In: *Proceedings of the 13th ACM International Conference on Bioinformatics, Computational Biology and Health Informatics*. BCB '22. New York, NY, USA, 2022:1–10.
2. Carson JL, Grossman BJ, Kleinman S, et al. Red Blood Cell Transfusion: A Clinical Practice Guideline From the AABB*. Annals of Internal Medicine 2012;157:49–58.
3. Eastridge BJ, Malone D, and Holcomb JB. Early predictors of transfusion and mortality after injury: a review of the data-based literature. The Journal of Trauma 2006;60:S20–25.
4. McCluskey SA, Karkouti K, Wijeysundera DN, et al. Derivation of a risk index for the prediction of massive blood transfusion in liver transplantation. Liver Transplantation 2006;12:1584–93.
5. Kuhne CA, Zettl RP, Fischbacher M, Lefering R, and Ruchholtz S. Emergency Transfusion Score (ETS): A Useful Instrument for Prediction of Blood Transfusion Requirement in Severely Injured Patients. World Journal of Surgery 2008;32:1183–8.
6. Walczak S and Scharf JE. Reducing surgical patient costs through use of an artificial neural network to predict transfusion requirements. Decision Support Systems. Decision Support for Health Care in a New Information Age 2000;30:125–38.
7. Walczak S. Artificial neural network medical decision support tool: predicting transfusion requirements of ER patients. IEEE Transactions on Information Technology in Biomedicine 2005;9:468–74.
8. Mitterecker A, Hofmann A, Trentino KM, et al. Machine learning–based prediction of transfusion. Transfusion 2020;60:1977–86.
9. Walczak S and Velanovich V. Prediction of perioperative transfusions using an artificial neural network. PloS One 2020;15:e0229450.
10. Karkouti K, Cohen MM, McCluskey SA, and Sher GD. A multivariable model for predicting the need for blood transfusion in patients undergoing first-time elective coronary bypass graft surgery. Transfusion 2001;41:1193–203.
11. Litmathe J, Boeken U, Feindt P, and Gams E. Predictors of homologous blood transfusion for patients undergoing open heart surgery. The Thoracic and Cardiovascular Surgeon 2003;51:17–21.
12. Goudie R, Sterne JAC, Verheyden V, Bhabra M, Ranucci M, and Murphy GJ. Risk scores to facilitate preoperative prediction of transfusion and large volume blood transfusion associated with adult cardiac surgery†. BJA: British Journal of Anaesthesia 2015;114:757–66.
13. Shah MD, Goldstein DP, McCluskey SA, et al. Blood Transfusion Prediction in Patients Undergoing Major Head and Neck Surgery With Free-Flap Reconstruction. Archives of Otolaryngology–Head & Neck Surgery 2010;136:1199–204.

14. Liu LP, Zhao QY, Wu J, et al. Machine Learning for the Prediction of Red Blood Cell Transfusion in Patients During or After Liver Transplantation Surgery. Frontiers in Medicine 2021;8:632210.

15. Chang SS, Duong DT, Wells N, Cole EE, Smith JA, and Cookson MS. Predicting blood loss and transfusion requirements during radical prostatectomy: the significant negative impact of increasing body mass index. The Journal of Urology 2004;171:1861–5.

16. Kadar A, Chechik O, Steinberg E, Reider E, and Sternheim A. Predicting the need for blood transfusion in patients with hip fractures. International Orthopaedics 2013;37:693–700.

17. Ho WH and Chang CS. Genetic-algorithm-based artificial neural network modeling for platelet transfusion requirements on acute myeloblastic leukemia patients. Expert Systems with Applications 2011;38:6319–23.

18. Krishna NM, Nagaraja P, Singh NG, et al. Evaluation of Risk Scores in Predicting Perioperative Blood Transfusions in Adult Cardiac Surgery. Annals of Cardiac Anaesthesia 2019;22:73–8.

19. Azur MJ, Stuart EA, Frangakis C, and Leaf PJ. Multiple imputation by chained equations: what is it and how does it work? International journal of methods in psychiatric research 2011;20:40–9.

20. Rafiei A, Moore R, Jahromi S, Hajati F, and Kamaleswaran R. Meta-learning in healthcare: A survey. arXiv preprint arXiv:2308.02877 2023.

21. Mahajan P, Uddin S, Hajati F, and Moni MA. Ensemble Learning for Disease Prediction: A Review. Healthcare 2023;11.

22. Rafiei A and Wang YK. Automated major depressive disorder classification using deep convolutional neural networks and Choquet fuzzy integral fusion. In: *2022 IEEE Symposium Series on Computational Intelligence (SSCI)*. IEEE. 2022:186–92.

23. Arora A, Alderman JE, Palmer J, et al. The value of standards for health datasets in artificial intelligence-based applications. Nature Medicine 2023;29:2929–38.

24. Rafiei A, Ghiasi Rad M, Sikora A, and Kamaleswaran R. Improving mixed-integer temporal modeling by generating synthetic data using conditional generative adversarial networks: A case study of fluid overload prediction in the intensive care unit. Computers in Biology and Medicine 2023:107749.

25. Lundberg SM and Lee SI. A Unified Approach to Interpreting Model Predictions. In: *Advances in Neural Information Processing Systems*. Ed. by Guyon I, Luxburg UV, Bengio S, et al. Vol. 30. Curran Associates, Inc., 2017:4765–74.
